# Coupling between prefrontal brain activity and respiratory sinus arrhythmia in infants and adults

**DOI:** 10.1016/j.dcn.2021.101047

**Published:** 2021-12-13

**Authors:** Trinh Nguyen, Stefanie Hoehl, Bennett I. Bertenthal, Drew H. Abney

**Affiliations:** a Department of Developmental and Educational Psychology, University of Vienna, Liebiggasse 5, 1010 Vienna, Austria; b Department of Psychological and Brain Sciences, Indiana University Bloomington, 1101 E. 10th St., Bloomington, IN 47405, United States; c Department of Psychology, University of Georgia, 110 Hooper Street, Athens, GA 30602, United States

**Keywords:** fNIRS, functional near-infrared spectroscopy, Respiratory-sinus arrythmia, fNIRS, Prefrontal cortex, Vagus nerve, Self-regulation, Brain-body connection

## Abstract

Self-regulation is an essential aspect of healthy child development. Even though infants are dependent on their caregivers for co-regulation during the first years, they begin to gain early regulatory abilities through social interactions as well as their own cognitive development. These early regulatory abilities continue to increase with the maturation of both the prefrontal cortex and the vagal system. Importantly, theoretical accounts have suggested that the prefrontal cortex and the vagal system are linked through forward and backward feedback loops via the limbic system. Decreased coupling within this link is suggested to be associated with psychopathology.

The primary goal of this study is to examine whether intrapersonal coupling of prefrontal brain activity and respiratory sinus arrythmia is evident in infancy. Using the simultaneous assessment of functional near-infrared spectroscopy and electrocardiography, we will use Cross-Recurrence Quantification Analysis to assess the coupling of prefrontal brain activity and respiratory sinus arrhythmia in 69 4–6-month-old infants and their mothers during rest.

Understanding the developmental emergence of the neurobiological correlates of self- regulation will allow us to help identify neurodevelopmental risk factors.

## Highlights

• In adults, self-regulation involves coupling between the prefrontal cortex (PFC) and vagal activity.
• We investigate whether the coupling of brain and respiratory sinus arrhythmia (RSA) activity is evident during infancy.
• Understanding the developing connection between the PFC and vagal system will help to elucidate the pathways involved in self-regulation.

## 1 Introduction

Self-regulation is a critical aspect of children’s socio-cognitive development. During the first years, infants depend on their caregivers to co-regulate when emotional challenge occurs (Porter, 2003). Still, infants show early regulatory abilities, as these abilities allow infants to bridge distressing instances of parents' unresponsiveness in their daily social interactions (Markova and Nguyen, 2021). Theoretical accounts have identified the prefrontal control of limbic regions (including the cingulate, insular cortices and the amygdala) as centrally involved in self-regulation (Ochsner and Gross, 2007, Perlman and Pelphrey, 2010). Moreover, the prefrontal cortex (PFC) is connected to the vagus nerve via feedforward as well as feedback connections (Beauchaine, 2015). One common index of vagal activity is respiratory sinus arrhythmia (RSA). Importantly, several forms of psychopathology are characterized by different types of PFC dysfunction, including both heightened PFC and reduced PFC responses (Maren et al., 2013, Menon, 2011), as well as low resting RSA and excessive or blunted RSA reactivity, which are peripheral biomarkers of poor executive control (Black et al., 2021, Thayer et al., 2009). Still, we know surprisingly little about the connection between the PFC and the striated vagus nerve striate in infants as well as adults and the role it plays for healthy neurophysiological development. In the present study, we will investigate the relation between the PFC and the vagal system in infants and their mothers during rest.

Human infants are not born with a fully developed myelinated vagus. Instead, vagal development continues in the first few months postpartum (Porges and Furman, 2011). Myelinated vagal fibers greatly increase between 30 and 32 weeks gestational age and six months postpartum (Sachis et al., 1982). During this time, vagal tone steadily increases and then stabilizes as the vagal fibers continue to increase (Izard et al., 1991, Porges and Furman, 2011, Porter et al., 1995). As the myelinated vagal fibers increase with development, visceral regulation improves, and this has been observed to be associated with infants starting to display enhanced behavioral and emotion regulation (Porges and Furman, 2011). Empirical evidence also reveals that during the first half year infants’ regulatory abilities are characterized by individual differences. At 4 months of age, only around 50% of infants show vagal suppression (RSA decrease) upon emotional challenge (Abney et al., 2021). The first half year after birth, therefore, represents a key period for the development of self-regulation through increasing vagal activity.

**Fig. 1:**
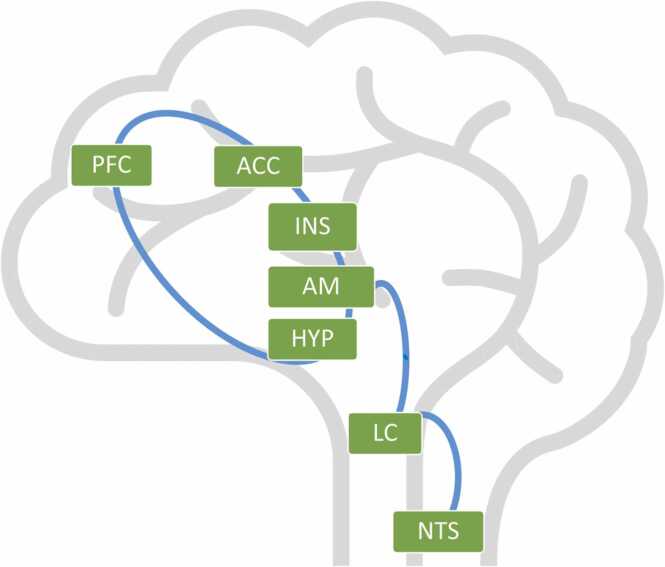
Schematic outline of the bidirectional connection between the prefrontal cortex (PFC), the anterior cingulate cortex (ACC), the insula (INS), the amygdala (AM) and the hypothalamus (HYP). The network is connected to the locus coeruleus (LC), which is connected to the nucleus solitary tract (NTS).

It is well established that older children and adults with attenuated self-regulation capacities (both excessive or dampened emotional reactivity dam or heightened distress/anxiety) often display low RSA tone during resting phases and excessive or blunted RSA reactivity to emotional challenge (Beauchaine, 2015, Black et al., 2021). Those individuals may display various forms of internalizing and externalizing psychopathology, and specific psychopathological syndromes, including anxiety, phobias, attention problems, autism spectrum disorder and depression (Beauchaine and Thayer, 2015). Emerging evidence suggests that low RSA and excessive/blunted RSA reactivity index poor self-regulation because they are downstream peripheral markers of PFC dysfunction. The medial prefrontal cortex (PFC) is connected to the parasympathetic nervous system via bidirectional pathways (see Fig. 1 for a schematic outline; Beauchaine, 2015). The prefrontal, cingulate, insular cortices, the hypothalamus, and the amygdala form a neural network (Thayer et al., 2009). The amygdala within this network is connected to the locus coeruleus, which in turn is connected to the nucleus solitary tract. These structures provide input via the parasympathetic nervous system to the sinoatrial node, which is the primary pacemaker of the heart (Porges, 1995). Taken together, cognitive appraisal modulates the pathways between the medial PFC and the parasympathetic nervous system, which can result in reduced basal RSA and excessive or blunted RSA reactivity in case of poor modulation (Beauchaine, 2015). Importantly, descriptions of these structural pathways between the PFC and the parasympathetic nervous system are based on adults, while it remains unclear if the structures can be applied to the infant biology. Still, even in adults, empirical evidence for the connection between the PFC and RSA is sparse. There is preliminary evidence, however, showing that heart rate variability is associated with amygdala-PFC functional connectivity and PFC activity during rest, underscoring neurovisceral involvement in self-regulation (Lane et al., 2009, Sakaki et al., 2016). However, to our knowledge, no prior work has examined the concurrent relation between PFC and RSA in children or infants. Accordingly, we assume that as the PFC and its functional connections develop throughout the first year of life, an association with the vagal system should increase. Brain maturation together with social experience could thus be associated with self-regulation, mediated by PFC-RSA coupling.

The PFC is related to numerous social and non-social cognitive functions and plays a fundamental role in understanding self and others (Frith and Frith, 2006). Previously, the PFC was assumed to be functionally silent during the first year of life. However, a growing body of research shows that functional PFC development and involvement is traceable from birth (e.g., Grossmann, 2013). Evidence suggests that the PFC is involved in various processes, such as speech and language, executive control and mentalizing. These processes are essential for social cognition and interaction. While the role of PFC in self-regulation at preschool age is well researched (Grabell et al., 2019, Perlman et al., 2014, Perlman and Pelphrey, 2010), there is less evidence regarding the involvement of the PFC in self-regulation during infancy. Still, for instance, cerebral blood flow in 5- to 8-month-old infants in frontal regions was positively related to temperamental negative affectivity and recovery of positive affect after a stressor (Catalina Camacho et al., 2020). These preliminary findings highlight the relevance of the PFC for self-regulation at both trait (i.e., temperament) and state level.

The first year of life plays an essential role in PFC development, as structural and functional alterations of the PFC become visible during this time. Premature birth, illness, or neglect may attenuate a healthy (neuro-) developmental trajectory (Porges and Furman, 2011). Atypical development could put infants at risk for lower sensitivity to social cues (see Grossmann, 2013) which is also modulated by the vagal system. The question then remains whether atypical development of either the PFC, the vagal system or both might inhibit the growing connection between the PFC and the striated vagal nerve, and consequently healthy child development. Infant temperament offers a look into early individual differences in affective and behavioral traits (Rothbart et al., 2011). More specifically, infants’ temperamental traits are defined in terms of a reactivity and regulation and are often assessed using parental reports as well as laboratory measures (Sidor et al., 2017, Tang et al., 2020). Accordingly, there is growing evidence suggesting a connection between temperament and RSA as well as PFC activity (Fox, 1989, Morales et al., 2015). For instance, connectivity in the aforementioned prefrontal, cingulate and insular network with the amygdala in newborns was associated with fear and cognitive development at 6 months (Graham et al., 2016, Thomas et al., 2019). Connectivity within this network has been specifically linked to infant temperament profiles, suggesting that a balance between negative affectivity and cognitive skills in infants allows them to efficiently self-regulate (Degnan and Fox, 2007, Nigg, 2006). We, therefore, propose variance in infants' coupling between PFC and RSA to be related to infant temperament. Understanding the PFC-vagal system vagal connection is of utmost importance to help identify and support infants at risk for developing difficulties in behavioral state regulation, poor affective tone, and diminished abilities for reciprocal social engagement behaviors.

### 1.1 Current study

**Fig. 2:**
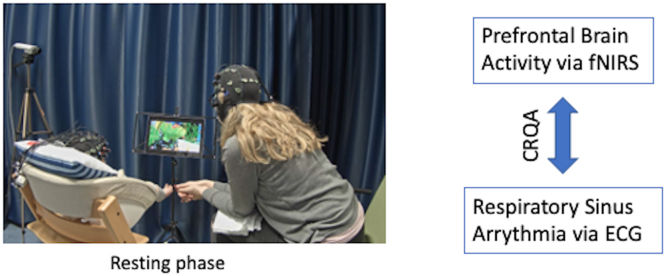
Exemplary dyad in the resting phase. Prefrontal brain activity and respiratory sinus arrythmia were concurrently measured in both infant and adult caregiver and we will assess whether the two time-series covary (using Cross-Recurrence Quantification Analysis [CRQA]) during rest (90 s). Created with BioRender.com.

The primary goal of this study is to examine covariation in PFC activity and vagal reactivity in infants at 4–6 months of age compared to an adult sample, comprised of infants’ mothers. We concurrently assessed PFC activity and vagal tone as assessed via RSA during a resting phase in both infant and caregiver (Fig. 2). Brain activity was continuously assessed using functional near-infrared spectroscopy (fNIRS) in medial and lateral PFC as well as bilateral inferior frontal gyrus. RSA was continuously assessed using electrocardiography and a novel dynamic sliding-window processing method. Our goal is to assess the association between brain activity and vagal tone in both infants and their adult caregivers over the course of a resting phase to assess if there is non-random coupling between RSA and PFC activity and if so, when this coupling develops. To do so, we will do the following:

We suggest that there will be covariation between PFC and RSA activity, meaning increases and decreases in PFC activity would be related to increases and decreases in RSA activity during a resting phase.

Next, we will examine whether and how coupling between PFC and RSA activity maps onto individual differences in infant temperament (negative affectivity, surgency and effortful control) as well as infant positive and negative affect during a free play situation with their mother. We hypothesize that higher PFC-RSA coupling is related to lower parent-reported negative affectivity, and higher surgency and effortful control. We also predict that higher PFC-RSA coupling is related to higher durations of positive affect and lower durations of negative affect during play.

We will also test whether this PFC-vagal system connection is related to individuals’ basal RSA. Here, we suggest that low basal RSA will be associated with decreased covariation between PFC activity and RSA reactivity, as the individuals’ RSA reactivity is restricted by the lower physiological boundaries of the vagal system.

Furthermore, we will investigate whether there is a developmental increase in the strength of this connection. More specifically, we predict that infants will show increased PFC and RSA covariation as they develop as a function of age.

## 2 Methods

### 2.1 Sample characteristics

Overall, 69 mother-infant dyads completed the experiment and were recruited from a database of volunteers. Infants’ age ranged from 4- to 6-months-old (*M* = 4.8 months; *SD* = 16 days; 33 girls). Infants were born healthy and at term, with a gestation period of at least 36 weeks. Mothers’ age averaged at 33.97 years (*SD* = 4.94) and 70.1% of mothers had a university degree. All dyads were of White European origin and came from middle to upper-class families based on parental education. All infants and mothers had no neurological problems as assessed by maternal report. An additional 11 infants participated in the present study but were excluded due to bad signal quality, fussiness (infants started to cry during the preparation phase or before the end of the experiment), and sleepiness. Included participants did not differ from the excluded participants in terms of child age, biological sex or maternal age and education, *p* > .051.

### 2.2 Experimental procedure

During the experiment, caregiver and infant were either seated next to one another (Fig. 2) or the infant sat on the caregiver's lap as both were watching a calm aquarium video on a tablet (resting phase and proximate watching condition). The videos lasted 90 s and depicted fish swimming in a tank. The order of the watching conditions was counterbalanced. Next, mother and infant engaged in a 5 min long free play without toys and song while both were seated face-to-face. For the purposes of this study, we consider the following experimental conditions: (1) the resting phase and (2) the free play condition to assess infant behavior. Neural activity in the mother-infant dyad was simultaneously measured with functional near-infrared spectroscopy. We assessed respiratory sinus arrhythmia (RSA) through electrocardiography (ECG) and each dyad was filmed by three cameras (angled towards the dyad, the infant, and the mother) throughout the experiment.

### 2.3 Data acquisition

#### 2.3.1 fNIRS Recordings

We used two NIRSport 8-8 (NIRx Medizintechnik GmbH, Germany) devices to simultaneously record oxy-hemoglobin (HbO) and deoxy-hemoglobin (HbR) concentration changes in mother and infant. The 8 × 2 probe sets were attached to an EEG cap with a 10–20 configuration (Fig. S1). The standard electrode locations allowed us to place the probes more precisely so that they were located over the left and right inferior frontal gyri (IFG) corresponding to F7 and F8, whereas the probes located over the medial prefrontal area (mPFC) corresponded to FP1 and FP2. These regions of interest were based on previous work involving adult-child interactions (Nguyen et al., 2020, Piazza et al., 2020, Redcay and Schilbach, 2019). In each probe set, 8 sources and 8 detectors were positioned, which resulted in 16 measurement channels with equal distances of ~ 2.3 cm between the infants’ optodes and 3 cm between the mothers’ optodes. The absorption of near-infrared light was measured at the wavelengths of 760 and 850 mm and the sampling frequency was 7.81 Hz.

### 2.4 Electrocardiogram (ECG) Recordings

We used a Brain-Amp system (Brain Products GmbH, Germany) with two amplifiers to measure two standard single-channel ECG registrations (lead II derivation). One electrode was placed on the upper right chest, one on the left side of the abdomen and the grounding electrode was placed on the right side of the abdomen on both infant and mother. The ECG signal was recorded with a 500 Hz sampling frequency. Interbeat-intervals (IBIs) were then extracted offline using ARTiiFACT (Kaufmann et al., 2011). The ECG data were visually inspected for (in)correct detections and artifacts by trained research assistants. When ectopic beats or erroneous detections were found, the data were manually corrected (removal of erroneous detection/artifact followed by a cubic spline interpolation; corrections < 1%).

### 2.5 Parent-reported infant temperament

Infant temperament was assessed by parent-report utilizing the very short form of the revised Infant Behavior Questionnaire (IBQ; Putnam et al., 2014). The questionnaire assesses three infant temperament subscales in 3–12-month-old infants. The subscales are surgency, effortful control and negative affectivity. The subscale surgency consists of items concerning the infants’ approach, vocal reactivity, high intensity pleasure, smiling and laughter, activity level, and perceptual sensitivity. The subscale negative affectivity consists of items regarding sadness, distress to limitations, fear, and rate of recovery from distress. The subscale orienting/regulation consists of items regarding low intensity pleasure, cuddliness, duration of orienting, and soothability (Gartstein and Rothbart, 2003). Mothers rated the frequency that their infant engaged in specific day-to-day behaviors in the prior one to two weeks using a 7-point scale, with responses ranging from 1 (never) to 7 (always).

### 2.6 Behavioral coding of infant affect

To assess infant affect, trained graduate students coded video recordings of the free-play sessions using Mangold INTERACT. The experimental sessions were filmed at 25 frames per second. Infant facial affect was micro-coded frame-by-frame for duration and frequency. We distinguished between positive (smiling with mouth turned upward (open or closed), contraction of cheek muscle and/or under-eye muscle), negative (expressing negative emotions: distress, fretting, anger, discontentment, sadness or “pout face” as indexed by narrowed eyes, mouth curled or grimacing, lowered brows, mouth corners turned down) and neutral facial expressions. We calculated inter-rater reliability in 25% of randomly chosen videos using kappa, which resulted in overall κ = .81 for facial affect. To control for minor variations in interaction duration (*M* = 291.36 s, *SD* = 38.08 s), the total duration of each facial affect category was divided by the total time of free-play (i.e., duration of coded free-play) to be able to control for different interactional durations between dyads. The affect scores thus indicate proportions of time during each condition.

### 2.7 Proposed analysis pipeline

fNIRS measurements were processed using MATLAB-based functions derived from Homer 2 (Huppert, Diamond, Franceschini, and Boas, 2009). Raw data were automatically pruned using the enprunechannels function (dRange = [0.03 2.5]; SNRThresh = 10) and subsequently converted into optical density. Next, optical density data was motion-corrected with a wavelet-based algorithm with an interquartile range of 1.5 (Molavi and Dumont, 2012). Motion-corrected time series were further visually inspected during a quality check procedure. We checked frequency-time plots of time-series for integrity, a clearly visible heart band, and visually observable motion artifacts (Nguyen, Hoehl et al., 2021) resulting in the removal of 22.87% of channels of infants and caregivers. Then slow drifts and physiological noise were removed from the signals using a band-pass second-order Butterworth filter with cutoffs of 0.01 and 0.5 Hz and a slope of 12 dB per octave. The filtered data were converted to HbO and HbR values (μMol) based on the modified Beer-Lambert Law. For the proposed secondary data analysis, HbO and HbR timeseries will be down sampled to 5 Hz to match the sampling rate of RSA.

IBIs were down sampled to 5 Hz and a 51-point band-pass local cubic filter was used to estimate and remove the slow periodic and aperiodic components of the time series (Abney et al., 2021). A FIRtype bandpass filter was applied to further isolate the variance in the IBI series to only the frequency range of spontaneous breathing for infants (0.3–1.3 Hz) and adults (0.12–1.0 Hz). The higher range of 1.0 Hz for mothers’ respiration is used to account for the infrequent occurrence of faster breathing during talking or playing segments so that the same filter could be used for all mothers in all conditions. The (Porges and Bohrer, 1990) technique for RSA magnitude estimation includes parsing this component signal into discrete epochs (lasting 10–120 s), then calculating the natural log of the variance in each epoch. RSA is reported in units of ln(ms)^2^. Instead of following the Porges and Bohrer method, we introduce a sliding window of 15 s that is used to extract a continuous (updated every 200 ms) estimate of cardiac vagal tone for both participants. The estimated RSA value corresponds to the first value of the sliding window. RSA values were then detrended by calculating the change values of RSA over the course of the distal watching condition (see Abney et al., 2021 for more details).

We plan to use Cross-Recurrence Quantification Analysis (CRQA) to calculate the coupling between prefrontal brain activity (HbO and HbR) in three different brain areas (IFG vs. lPFC vs. mPFC) and RSA change values (Coco and Dale, 2014; Konvalinka et al., 2011). Coupling will be calculated in the distal watching condition, for which 90 s of data is available from each included infant and adult. CRQA is a nonlinear method for analyzing shared dynamics between two different data series (Shockley et al., 2002) and has been applied successfully to investigate cardio-respiratory dynamics (Konvalinka et al., 2011, Nguyen et al., 2021) and behavioral coordination (Abney et al., 2015). The method is especially suitable for neurophysiological coupling estimations, as it does not assume stationarity within the data. Broadly, CRQA provides information about how often two signals co-visit areas in a state-space that is inferred from the measured time series. When two signals co-visit the same areas of a state-space at approximately the same time, this increased temporal coordination is reflected in high recurrence rates. We can also investigate long-range patterns of influence across two signals using CRQA by quantifying the similarity of signal patterns that are separated in time.

The metric we plan to use to evaluate neurophysiological coupling is the diagonal-wise cross-recurrence rate (REC) of the two continuous standardized neural and physiological signals from each infant and adult. We will utilize the drpdfromts function from the crqa package (Coco and Dale, 2014) and set the window size to ± 10 s and radius to.02. For the statistical analyses, we will take the maximum recurrence observed (MAXREC) into account. Statistical analyses on neurophysiological coupling will focus on HbO and RSA values, while coupling analyses between HbR values and RSA will be included in the Supplemental materials.

Control analysis. We will compare REC between observed neurophysiological coupling and random pairings of neural and physiological time series of participants to assess the degree of neurophysiological coupling. By establishing a baseline of coupling through surrogate analysis (e.g., Abney et al., 2015; Nguyen, Abney, et al., 2021; Nguyen, Hoehl, et al., 2021), we can compare properties of the observed PFC-RSA coupling against what might be expected by chance / spurious correlation. For the surrogate test, we will create surrogate data series combinations by randomly pairing the neural time series of each participant with the RSA time series of other participants. This provides a baseline for the rates of recurrence that could be expected by chance or the amount of similarity of behavior that we might expect as a function of the environment or task rather than the actual process of coupling. We will create 400 non-repeated surrogate pairs and then perform CRQA on each pairing to estimate recurrence profiles. The control analysis will be conducted for the infant and adult sample, respectively.

To statistically compare the rates of recurrence between observed coupling to random surrogate coupling, we will perform generalized linear mixed effects modeling. Two separate models will be estimated for infants (Model 1) and adults (Model 2). MAX REC will be added as the response variable and modeled over fixed effects of pairing (true vs. random), region (IFG vs. lPFC vs. mPFC) and the interaction between pairing and region. Random slopes will be added for pairing and region, and random intercepts will be assumed for individuals. We can therefore conclude whether the observed levels of PFC-RSA covariation, as viewed through CRQA, are significantly above spurious covariation. In case the model estimation does not converge, we will remove the random slopes in the following order: region, pairing.

The following covariates will be considered. Firstly, infant temperament (i.e., negative affectivity, surgency, effortful control), assessed by parent-report and infant positive and negative affect during the free play condition will be tested as covariates in PFC-RSA coupling. Secondly, basal RSA will be estimated by calculating the average RSA over the distal watching condition. Next, infants age in days will be included. To test the covariates, we will run three additional models. Model 3 will the test the effects of infant temperament and behavior on infants intrapersonal coupling of PFC and RSA activity. MAX REC will be added as the response variable and modeled over fixed effects of region, infant negative affectivity, proportionate durations of infants’ positive and negative affect. Model 4 will test the effects of basal RSA, and infant age on PFC-RSA coupling. MAX REC will be added as the response variable and modeled over fixed effects of region, infant basal RSA and infant age. In case Model 1 displays regional differences in intrapersonal PFC-RSA coupling, we will include interaction effects between region and the covariates, respectively. Model 5 will the test the effects of the adult basal RSA on adults intrapersonal coupling of PFC and RSA activity. MAX REC will be added as the response variable and modeled over fixed effects of region and basal RSA. Again, in case Model 2 displays regional differences in intrapersonal PFC-RSA coupling, we will include interaction effects between region and basal RSA. In all models, random slopes will be added for region as well as covariates (Model 3: negative affectivity, negative affect, positive affect; Model 4: infant basal RSA, infant age; Model 5: adult basal RSA), and random intercepts will be assumed for individuals. In case the model estimation does not converge, we will remove the random slopes in the following order: region, Model 3: positive affect, negative affect, negative affectivity; Model 4: infant age, basal RSA; Model 5: adult basal RSA.

### 2.8 Previous author involvement

Author T.N. was involved in data collection, preprocessing and previous analyses (analyses on interpersonal neural and physiological synchrony as well as maternal touch), will thus provide author D.H.A. with fNIRS and ECG pre-processed data to ensure unbiased data analyses. All following data and statistical analyses will be conducted by D.H.A., who was not involved in data collection and previous analyses. All authors were involved in writing a manuscript concerning the role of proximity and touch in neural and physiological synchrony in naturalistic mother-infant interaction.

### 2.9 Statistical Power Analysis

We conducted a statistical power analysis using a bespoke web application (Judd et al., 2017). We entered a sample size of 65, considering further dropouts due to low data quality. Previous research concerning neural correlates of heart rate variability measures displayed large effect sizes (d > 0.8) (Lane et al., 2009, Sakaki et al., 2016). Therefore, we conducted the power analysis assuming large and moderate effect sizes (d = 0.5–0.8), which resulted in 1-β = 0.871–0.999. Accordingly, our sample size is appropriate for the planned analysis.

### 2.10 Timeline

If Stage 1 review is successful, data analysis will start in the winter term of 2021/2022. Following this, preparation of the full manuscript will occur between March and August of 2022. The full manuscript will be submitted in November 2022.

## Ethics approval statement

Ethics approval was granted by the ethics committee of the University of Vienna (00352).

## Funding statement

The study was funded by a professorial starting grant by the 10.13039/501100003065University of Vienna (S.H.) and by a stipend by the Studienstiftung des deutschen Volkes (T.N.).

## Data Statement

Raw and processed data will be made available upon Stage 2 Submission. Analyses scripts are made available on the online repository OSF: https://osf.io/sr6hf. Following Stage 1 in principle acceptance, we agree to register their approved protocol on the Open Science Framework (http://osf.io/rr/). If we later withdraw the paper, we agree to the Journal publishing a short summary of the pre-registered study under a section Withdrawn Registrations.

## Declaration of Competing Interest

The authors declare that they have no known competing financial interests or personal relationships that could have appeared to influence the work reported in this paper.

## Data Availability

fNIRS and RSA data will be made available on Registered Report Stage 2 submission. Data processing and analysis scripts are made available on OSF: https://osf.io/sr6hf.
