# Supplementary material for: Coupling between prefrontal brain activity and respiratory sinus arrhythmia in infants and adults
Source: Dev Cogn Neurosci. 2021 Dec 13;53:101047. doi: 10.1016/j.dcn.2021.101047 (PMC8703057; doi:10.1016/j.dcn.2021.101047)
Supplement: Supplementary file 1 — Supplementary material [file mmc1.docx]

Supplementary Information to manuscript:

“Coupling between Prefrontal Brain Activity and Respiratory Sinus Arrhythmia in Infants and Adults”

Supplementary Figures

*
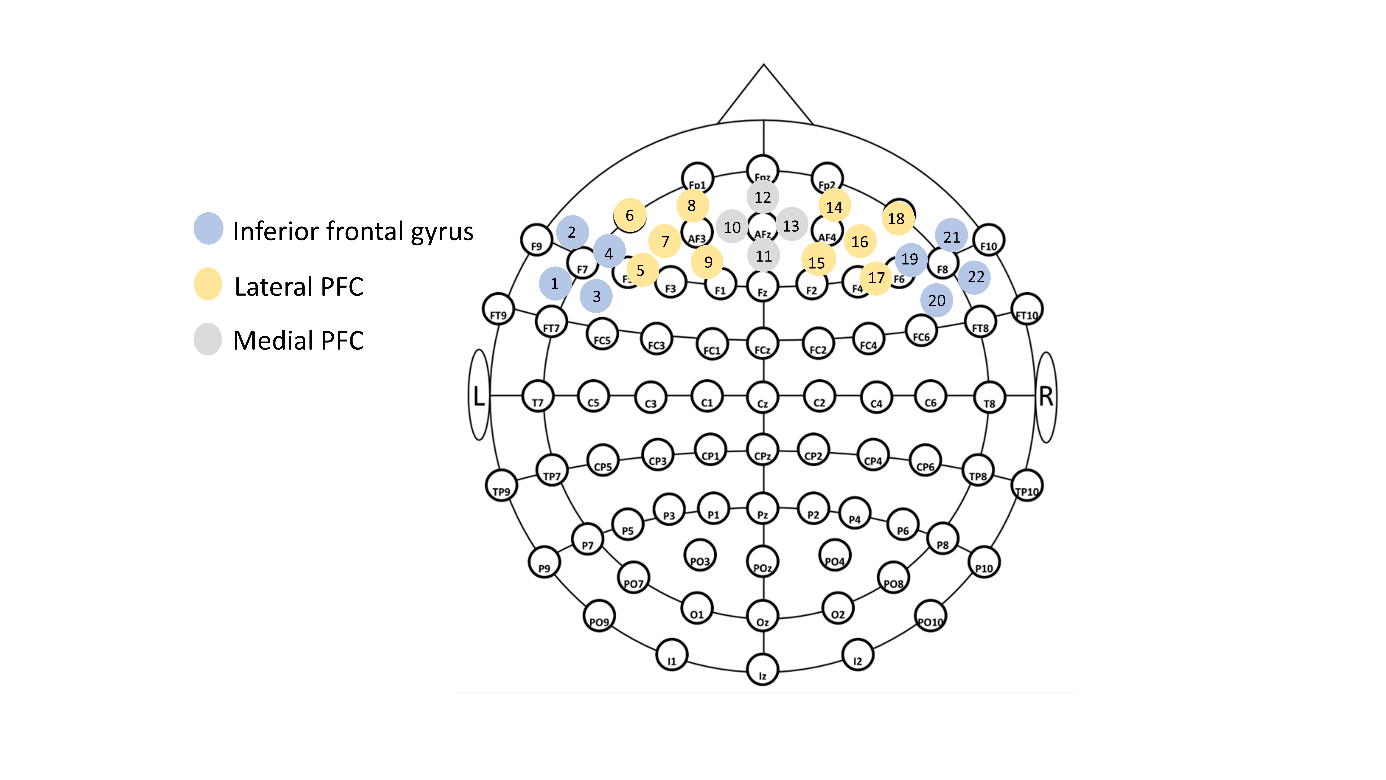
*

*Figure S1.* The channel configuration of the mother and infant cap comprises the following brain areas: Inferior frontal gyrus (1-4; 19-22), lateral prefrontal cortex (PFC; 5-9; 14-18), and medial prefrontal cortex (10-13).


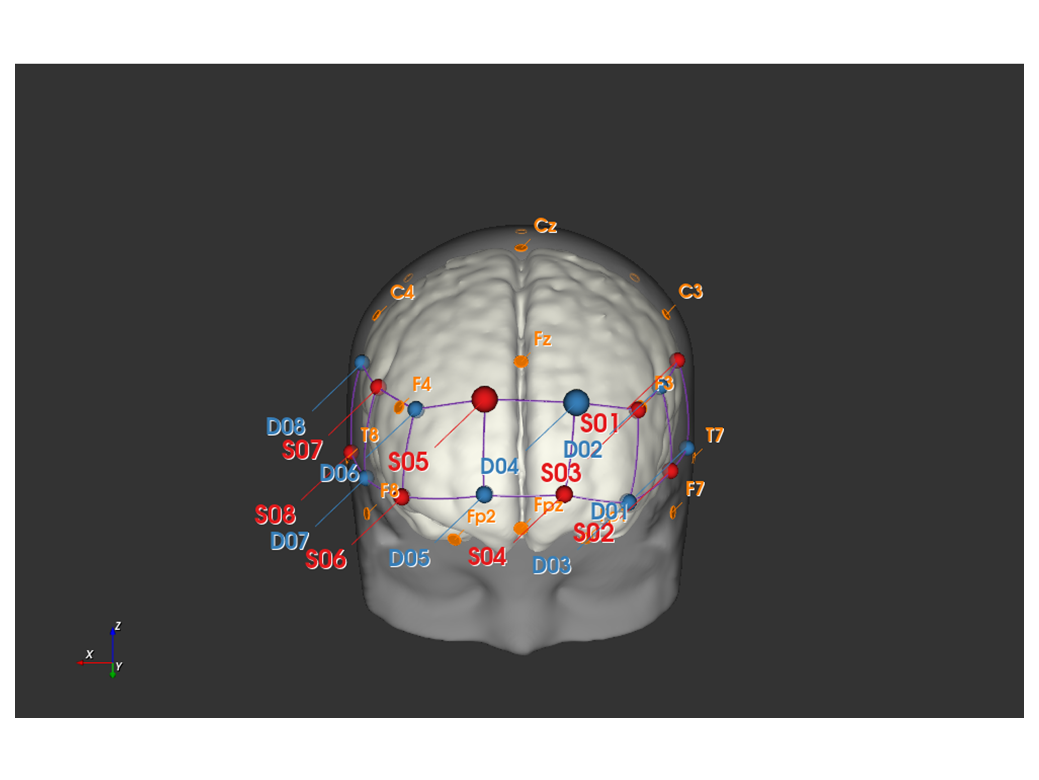

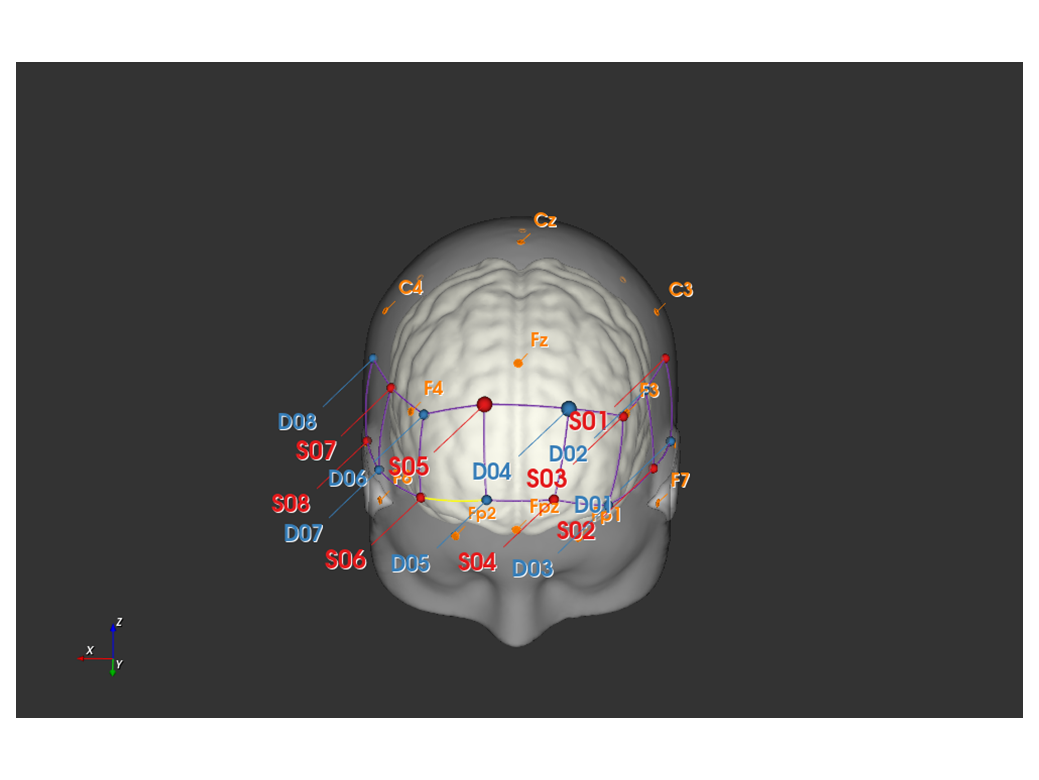


*Figure S2.* Optode positions are projected onto a 2-5-month-old infant head model (Infant Atlases 0-4.5 years; top) and an adult head model (ICBM 152 Nonlinear atlases version 2009; bottom). EEG 10/20 reference labels, which we used to place optode positions onto the caps, are marked in orange. Sources are in red, detectors in blue. Projections were implemented using NIRSite 2021.4.
